# Development of a Conceptual Model for the Patient Experience of Focal Segmental Glomerulosclerosis (FSGS): A Qualitative Targeted Literature Review

**DOI:** 10.1007/s12325-023-02651-6

**Published:** 2023-10-11

**Authors:** Natalie V. J. Aldhouse, Helen Kitchen, Tamara Al-Zubeidi, Madeleine Thursfield, Randall Winnette, Sandi See Tai, Linda Zhu, Nicolas Garnier, Christine L. Baker

**Affiliations:** 1 Clinical Outcomes Assessment, Clarivate, London, UK; 2 Pfizer Inc, New York, NY USA; 3 Pfizer Inc, Collegeville, PA USA

**Keywords:** Clinical outcomes assessments, Focal segmental glomerulosclerosis, Patient experience, Patient-reported outcomes, Qualitative

## Abstract

**Introduction:**

Focal segmental glomerulosclerosis (FSGS) is a leading cause of kidney disease and can progress to end stage kidney disease (ESKD). An overview of symptoms and impacts of the disease experienced will help inform the selection or development of fit-for-purpose clinical outcome assessments (COA) to be used in FSGS clinical trials. This study aimed to develop a conceptual model (CM) of the adult and pediatric patient experience of FSGS including disease signs/symptoms, treatment side-effects, and impact on functioning and wellbeing.

**Methods:**

This study comprised a systematic review and thematic analysis of qualitative studies with adults and pediatric patients diagnosed with FSGS. Data sources were identified through an electronic database search of journal articles (Medline, Embase, PsycINFO; June 2021) and hand-searching of conference proceedings, patient advocacy group websites, and gray literature. Non-English articles were excluded. Identified data (patient/caregiver quotes, author summaries, and interpretations of patient experiences) were extracted from the articles. Extracted data were qualitatively analyzed aided by ATLAS.ti v7. Codes were applied to data and concepts (symptoms/impacts) were identified, named, and refined. A CM was developed by grouping related concepts into domains.

**Results:**

In total, 12 sources were identified for analysis: 6 journal articles and 6 series of patient testimonials. Salient sign/symptom/side-effect domains included swelling/puffiness (edema), pain/aches/discomfort, fatigue, weight changes, skin problems, respiratory problems, and sleep problems. Salient impact domains included emotional/psychological wellbeing, physical functioning/activities of daily living, social functioning, and work/school.

**Conclusion:**

Secondary analysis of published qualitative literature permitted development of a CM describing the adult and pediatric experience of FSGS. Concept elicitation interviews are recommended to refine the CM, confirm the salient/most bothersome concepts, and confirm the extent of impact on daily life. The refined CM will provide a useful tool to inform the selection, development, and/or amendment of COAs for use in future FSGS clinical trials.

**Supplementary Information:**

The online version contains supplementary material available at 10.1007/s12325-023-02651-6.

## Key Summary Points

**Table:** 

A conceptual model describing the adult and pediatric experience of focal segmental glomerulosclerosis (FSGS) was developed from a secondary analysis of published qualitative literature.
The model provides a useful tool to inform the selection and/or development of clinical outcome assessments for use in future FSGS clinical trials.
Salient sign/symptom/side-effect domains included swelling/puffiness (edema), pain/aches/discomfort, fatigue, weight changes, skin problems, respiratory problems, and sleep problems.
Salient impact domains included emotional/psychological wellbeing, physical functioning/activities of daily living, social functioning, and work/school.
Concept elicitation interviews are recommended to refine the model, explore the salient/most bothersome concepts, and confirm the extent of the impact on the daily lives of patients.

## Introduction

Focal segmental glomerulosclerosis (FSGS) is a leading cause of kidney disease and can progress to end stage kidney disease (ESKD) [1]. Characterized by histopathologic lesions which develop in the glomeruli, patients with FSGS often present with nephrotic syndrome, before FSGS is diagnosed through kidney biopsy [2]. FSGS accounts for approximately 40% of adults with nephrotic syndrome (NS) [3] and approximately 20% of cases of NS in children. Reviews suggest the incidence of FSGS in the US may range from 1.4 to 21 cases per million [4]. However kidney biopsies are less commonly performed in children than in adults, and it is therefore difficult to estimate the total prevalence of FSGS in children [2].

FSGS can be classified into primary, genetic, and secondary causes which may include medications, viral infections, or other conditions such as obesity, certain renal anomalies, diabetes mellitus [4, 5], lupus erythematosus [5, 6], and amyloidosis [5]. Currently, there are no US Food and Drug Administration (FDA)-approved disease-modifying treatments for FSGS [7]. Treatment varies depending on the cause and type of FSGS, but often involve immunosuppressive therapy, such as corticosteroids, particularly in primary FSGS, and strict diet change [8]. However, corticosteroid treatment is associated with considerable burden, including side effects [7]. While most patients respond to corticosteroid treatment, some become resistant to treatment and progress to ESKD [8]. FSGS can occur at any age [9], and has a substantial impact on health-related quality of life (HRQoL); patients report impairment in aspects of daily life and emotional wellbeing [10]. Additionally, patients may live with the prospect of kidney failure and rapid progression to ESKD, requiring dialysis or a kidney transplant [11]. FSGS can also reoccur after transplant [12], and patients may need consecutive transplants. Consequently, there is considerable unmet need in the treatment of FSGS. To address this need, several potential treatments are under investigation in clinical trials across various stages of development [7].

It is therefore important to understand the patient experience of FSGS to identify the symptoms and impacts of the disease that are most important for treatments to target. Indeed, initiatives, such as the Standardized Outcomes in Nephrology-Glomerular Disease (SONG-GD), are underway to establish a core outcome set for trials in adults with glomerular disease [13, 14]. As part of the initiative, important outcomes for measurement have been identified including outcomes that can be measured clinically, such as kidney function, mortality, and need for dialysis or transplant, and outcomes that can be reported by patients, such as life participation, fatigue, anxiety, family impact, and ability to work.

Obtaining patient insights to inform the selection and/or development of fit-for-purpose clinical outcome assessments (COAs) to be included in clinical trials is in accordance with the FDA Patient Reported Outcome (PRO) Guidance for Industry [15] and the Patient-Focused Drug Development final and draft guidance documents [16, 17]. A first step in identifying fit-for-purpose COAs is to conceptualize the disease experience through collecting patient experience data [18].

The objective of this study was therefore to conduct a qualitative literature review to understand the patient experience of FSGS and to develop a conceptual model for the disease, so that future clinical trials can be designed to report on outcomes that matter most to patients.

## Methods

### Selection Criteria

Qualitative studies using methods such as qualitative interviews or focus groups to explore the patient experience of FSGS or primary NS were identified via a targeted literature review and screened using pre-defined eligibility criteria (Table 1). No ethics approval was sought due to this being a secondary synthesis of data from published sources that are publicly available and/or from previous studies where ethics approvals were in place. This article is based on previously conducted studies and does not contain any new studies with human participants or animals performed by any of the authors.

**Table 1:** Study selection eligibility criteria

Criteria	Include	Exclude
Population	Patients of any age or gender diagnosed with FSGS and/or primary NS Parents/caregivers of children/adolescents diagnosed with FSGS and/or primary NS	Patients with secondary NS, e.g., caused by diabetes mellitus, lupus erythematosus, amyloidosis, etc.
Intervention/comparator	No restrictions	No restrictions
Study design	Qualitative interviews or focus groups	Studies with quantitative methodology only
Outcomes	Patient experience of FSGS/primary NS: Patient/caregiver descriptions of symptoms, side effects, and HRQoL of FSGS Outcomes reported as direct quotes or author summaries of collected data	Quantitative results only (e.g., COA measure scores)
Publication language	English language Highly relevant studies with English language abstracts but non-English language manuscripts were identified for potential translation	Publications with non-English language abstracts
Date of publication	No restrictions	No restrictions

*COA* clinical outcome assessment, *FSGS* focal segmental glomerulosclerosis, *HRQoL* health-related quality of life, *NS* nephrotic syndrome

### Data Sources and Searches

A detailed search strategy protocol was developed and included several data sources. Electronic database searches were run in Medline (1946–present), Embase (1980–present), and PsycINFO (1967–present) via Ovid in June 2021 to identify relevant publications. The search strategies are provided in Tables S1–S3 in the electronic supplementary material. Hand-searching of relevant FDA and European Medicines Agency meetings/workshops/forums, the patient advocacy group Nephcure’s website and YouTube channel, the reference lists of included studies/relevant reviews from the database search and abstracts of three kidney conference proceedings (American Society of Nephrology: The Kidney Week; European Renal Association – European Dialysis and Transplant Association Congress; International Society of Nephrology): The World Congress of Nephrology) (2019–2021) was conducted. Two authors (TaZ and NVJA) screened the titles and abstracts (first pass). Non-relevant studies were excluded, and potentially relevant studies were retrieved for full publication review (second pass).

### Quality Assessment

Each qualitative study was independently assessed by authors TaZ and NVJA using the Critical Appraisal Skills Programme qualitative checklist [19]. Low-quality studies were flagged for potential exclusion if deemed likely to contain inaccurate and/or unreliable results. Any discrepancies were resolved by discussion.

### Data Extraction and Analysis

Extracted patient/caregiver quotes or author descriptions/interpretations underwent primary (for the interview transcripts) or secondary (for the publications identified in the literature review) analysis using inductive, semantic thematic analysis techniques aided by ATLAS.ti Version 7. Author TaZ led coding and identified patient experience concepts. Authors TaZ, NVJA, MT, and HK grouped concepts into themes and sub-themes. TaZ drafted a conceptual model, a pictorial representation of the patient experience of FSGS positing groupings and links between concepts, with each component of the conceptual model supported by illustrative quotes. The conceptual model was refined through discussions with all other authors. Concepts reported only by patients with primary NS were marked on the conceptual model.

## Results

### Study Selection Summary

**Fig. 1:**
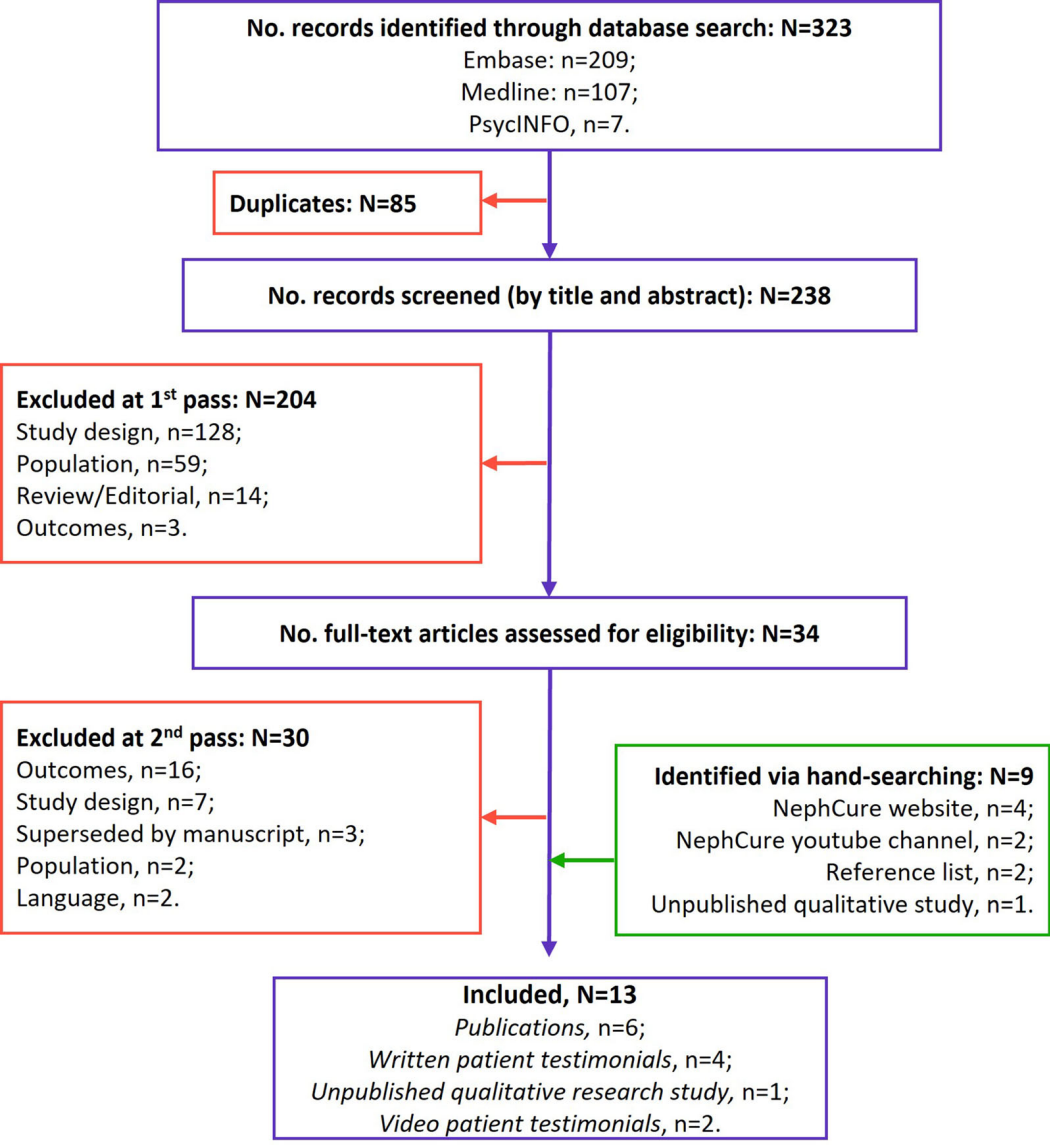
Literature review PRISMA flow diagram. *No.* number

A total of twelve studies were included for review: six published qualitative research studies [10, 11, 20–23]; four written patient testimonial series from a patient advocacy group (Nephcure) [24–27], and two video patient testimonials from a patient advocacy group (Nephcure) [28, 29]. The 13 studies explored FSGS (*n* = 7) and NS (*n* = 6). Six studies included an adult population [11, 21, 22, 24, 25, 28], four studies included a pediatric population [23, 26, 27, 29], and two studies included both adult and adolescent populations [10, 20]. All studies were conducted in the US or Canada. The included studies are summarized in Table S4 in the electronic supplementary material. The process of study selection is documented in the Preferred Reporting Items for Systematic Reviews and Meta-Analyses (PRISMA) [30] flow diagram in Fig. 1; no studies were excluded due to quality assessment results.

### Conceptual Model of the Patient Experience of FSGS

**Fig. 2:**
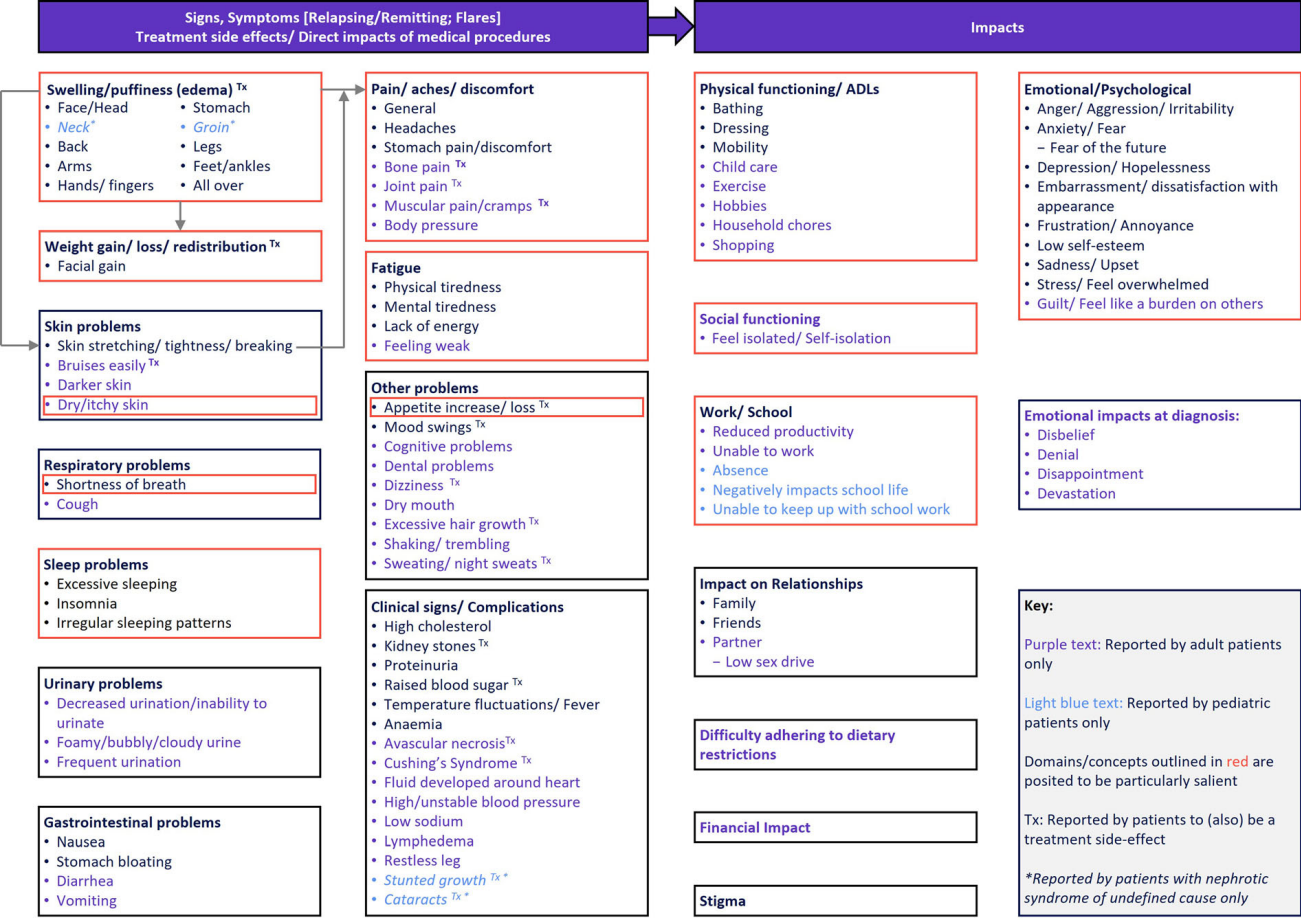
Conceptual model of with the patient experience of FSGS/NS. *FSGS* Focal segmental glomerulosclerosis, *NS* nephrotic syndrome

A conceptual model of the patient experience of FSGS is presented in Fig. 2. The conceptual model presents FSGS signs/symptoms, treatment side-effects, and their impact on patients’ functioning and wellbeing. Arrows indicate where there is a posited relationship or interaction between concepts. An existing CM was identified in the published literature [11] and reviewed alongside other evidence. Concepts are organized into related domains and concepts reported by a sub-sample only are noted. Domains and concepts outlined in red are posited to be particularly salient. Some physical experiences were described as a disease sign/symptom and a treatment side effect. Almost all concepts were reported by patients with FSGS; ‘cataracts’ and ‘stunted growth’ were the only concepts reported by patients with NS only.

### Concepts

#### Signs, Symptoms, Treatment Side-Effects, Direct Impacts of Medical Procedures

The literature documented that FSGS has a relapsing/remitting profile. Patients reported that symptoms were rarely stable across time, describing unpredictable ‘flare-ups’, which caused them both annoyance and concern [10, 11, 20, 24–27, 29].

##### Swelling/Puffiness (Edema)

Swelling was the most commonly reported symptom of FSGS, reported by both adult and pediatric patients, and often the symptom that triggered a search for a diagnosis [10, 11, 20, 22, 24–29]. Swelling was reported as occurring across all areas of the body, but most frequently in the lower limbs, face/eyes, and stomach/abdomen.

Swelling caused weight gain and skin problems as the skin stretched over the accumulated fluid; in some cases, the skin would break. Swelling was therefore commonly associated with reports of feelings of pain [10, 11, 27, 28], hurt [10, 27], discomfort [10], tightness [11], and pressure due to pressure on the body [10], the stretching of the skin [11, 27, 28], or constriction caused by too-tight clothing, many patients reported difficulty getting dressed and finding appropriate clothing [10].

Swelling could have a significant impact on patients’ appearances and, subsequently, cause emotional discomfort; patients reported feeling embarrassment and self-consciousness when around others, particularly if the swelling was visible in their face, or if it made them appear overweight [10, 26, 28].

##### Weight Gain/Loss/Redistribution

In addition to the weight gain associated with swelling, changes in weight were attributed as a side effect of corticosteroid treatment [10, 22, 25].

##### Skin Problems

Skin problems were not solely caused by swelling. Dry/itchy skin was reported in one publication, and named as a particularly bothersome experience that could make it difficult to sleep [11]. It was also reported that corticosteroid treatment could cause skin changes, such as sun sensitivity [29], susceptibility to bruising [11], and darker skin [11].

##### Respiratory Problems

Patients reported experiencing shortness of breath, typically during daily physical activities such as climbing stairs, and this could induce feelings of panic [10, 11, 22]. Coughing was also reported in two papers, but not in sufficient detail to provide information regarding the type of cough experienced [11, 22].

##### Sleep Problems

Sleep problems were commonly reported. Most frequently, patients reported difficulty sleeping or difficulty falling asleep, disrupted sleep with frequent awakenings, or sleep that did not make the patient feel restful when they awoke [10, 11, 22, 25, 27]. Difficulty sleeping was commonly associated with corticosteroid treatment, and could be unprovoked or caused by pain or itchy skin. Conversely, patients also reported sleeping ‘too much,’ in association with feelings of fatigue [10, 25].

##### Urinary Problems

Urinary problems were a key component of FSGS, with patients reporting both reduced urination/an inability to urinate [11, 26, 28], and foamy/bubbly/cloudy urine [11, 22, 25]. Conversely, patients also reported the need to frequently urinate, commonly following the onset of corticosteroid treatment, [11, 22, 24, 28].

##### Gastrointestinal Problems

Patients reported various gastrointestinal problems [10, 11, 25, 27]. Nausea was often reported as an initial symptom that prompted the search for a diagnosis and also a side effect of treatment [10, 11, 25, 28], and may be associated with vomiting [11, 25, 26, 28]. Nausea and vomiting made it difficult for patients to eat, and to perform some daily activities. Patients also reported stomach bloating, although the literature was unclear whether this was the same or different than the reported abdominal swelling/edema [22, 27], and diarrhea, which was associated with medication [11, 25].

##### Pains/Aches/Discomfort

Patients often reported experiencing pains [10, 11, 24–28], aches, and discomfort. Pain seemed to be caused by multiple factors. In addition to the pain associated with swelling/edema and skin problems (see previous section), patients reported stomach hurt/pain [24, 27, 29], muscular hurt [10], and bone pain. ‘Aches’ were associated with headaches [10, 27] or stomach aches [29], and ‘discomfort’ was associated only with swelling/edema [10].

##### Fatigue

Patients experienced fatigue as a multifaceted concept; feeling sleepy and tired [10, 11, 25, 26], low energy [10, 11, 22, 27], weak [22], and mentally/cognitively fatigued, experiencing ‘brain fog’ or difficulty thinking clearly [10]. Patients found it difficult to perform their daily activities, be productive at work, or attend social events, and, to compensate, some patients reported spending time resting/sleeping [10, 11, 27].

##### Other Problems

Patients also reported various other symptoms/treatment side effects which were not grouped into domains. These included changes in appetite [10, 11, 25, 29], mood swings [10, 11, 22, 28], cognitive problems [10, 11], dizziness [10, 11, 25], shakiness/trembling [11, 27], and sweating/night sweats [10, 11]. Many of these experiences were attributed as a side effect of corticosteroid treatment. Dental problems [11], dry mouth [11], and excessive hair growth [22] were also named in the literature, but only in one publication and without any description or patient quotes.

##### Clinical Signs/Complications

In addition to the patient-reportable signs and symptoms of FSGS, and side effects of treatments for FSGS, patients described various clinical signs or complications they learned they were experiencing from interactions with their clinicians. These included the key sign of proteinuria [11, 20, 25–27], and also high cholesterol [25, 27], kidney stones [22, 26], raised blood sugar [25, 27], temperature fluctuations/fever [10, 20, 27], Cushing’s syndrome [25], high/unstable blood pressure [20, 25–27], restless leg [22], stunted growth [26, 29], and cataracts [26].

##### Differences Between Adult and Pediatric Patients

Figure 2 indicates the signs, symptoms, symptoms, treatment side effects, and complications reported by adult patients only, pediatric patients only, and both. Signs and symptoms reported by adults only included skin bruising, darker skin, dry/itchy skin, cough, urinary problems (decreased urination, foamy/bubbly/cloudy urine, and frequent urination), diarrhea, vomiting, bone pain, joint pain, muscular pain/cramps, body pressure, feeling weak, cognitive problems, dental problems, dizziness, dry mouth, excessive hair growth, shaking/trembling, sweating/night sweats, and several clinical signs/complications. Signs and symptoms reported by pediatric patients only included swelling (edema) in the neck and groin, and two clinical signs/complications (stunted growth and cataracts).

#### Impact on How Patients Feel and Function

Patients reported that FSGS impacted how they felt and functioned. Concepts were grouped into domains that described impacts on physical function and activities of daily living (ADLs), social functioning, work/school, relationships, diet, emotional/psychological functioning, stigma, and finances.

##### Physical Functioning/ADLs

Patients reported that FSGS significantly impacted their daily lives. Symptoms such as swelling, fatigue, and nausea limited patients’ energy and caused pain, which made it difficult to complete household chores [10, 11], shop [10, 22], eat [10, 22], perform self-care [10, 22, 24], or look after their children [25]. As discussed in the section on swelling, patients found it difficult to dress when clothing no longer fit due to their changed body shape.

Symptoms, particularly swelling, but also dizziness and pain, also caused difficulties with mobility and impacted physical function. Patients found it difficult to walk [10, 11, 25, 26], use stairs [10, 22, 25], or even stand [28]. Patients were unable to exercise [10], and had to limit participation in their hobbies [10, 11, 26].

##### Work/School

Patients described times when they were unable to work or go to school due to their FSGS symptoms or medical appointments [10, 29]; for some patients, they had to stop working completely [10, 11, 24, 25, 28]. When patients did attend work/school, they described lower productivity and/or difficulty keeping up with the work, either due to previous time missed or because of cognitive symptoms that made it difficult to concentrate [10, 11, 22]. For children at school, absences also had a social impact, as they missed events they wished to attend or because their friendships were affected [10, 23].

##### Social Functioning

Patients often limited their social interactions due to their FSGS, either because they felt unwell, were self-conscious about their appearance, or because they needed to spend time in hospital [10, 11, 22, 26, 27]. This often caused patients to feel isolated [10, 11, 25, 29], which was compounded by feelings that nobody else could understand their experiences [21, 26].

##### Relationships

Patients reported that FSGS could negatively impact relationships with friends and family [10, 11, 23, 25, 29]. As discussed in previous sections, symptoms such as swelling, fatigue, pain, and associated reduced mobility sometimes prevented patients from leaving home to socialize and spend time with family members. Low sex drive was also reported as a symptom of FSGS in one publication [11], although no patient description of this was provided.

##### Dietary Restrictions

Patients often reported making alterations to their diet, including reduced fluid intake, in order to accommodate their FSGS [10, 20, 25–29].

##### Financial Impact

Patients with FSGS reported experiencing subsequent financial impacts [10, 11], which arose from being unable to work due to symptoms, and from the cost of health insurance and treatments.

##### Emotional/Psychological and Stigma

Diagnosis with FSGS was associated with feelings of disbelief and denial for some patients [11, 20, 28], and additional emotional/psychological impacts were often experienced throughout patients’ disease journeys.

Patients described feeling angry, frustrated, and stressed [10, 11, 20–22, 25], mainly due to the unpredictability of FSGS and the lack of treatment options, which made it hard for patients to plan their lives. Reports of anxiety and fear were common, ranging from acute panic attacks to chronic anxiety, as patients worried about medical appointments, the difficulty of finding efficacious treatment, that they would relapse, that they would deteriorate and require dialysis or a transplant, or simply due to the uncertainty of the future [10, 11, 20–22, 25, 26, 28]. Some patients felt guilty that their illness made them a burden on others [25].

Patients also reported feelings of depression, hopelessness, and sadness [10, 11, 22, 24, 25, 27, 28], often related to swelling and weight gain, or because FSGS symptoms or treatment side effects prevented them from doing the things they wanted to do. The overall experience of living with FSGS could also cause these feelings, however, due to the numerous impacts caused and the overall difficulty of managing an unpredictable condition.

As discussed, some patients expressed embarrassment [10, 28] and low self-esteem [10, 25] owing to change in appearance caused by swelling and weight gain. This could also cause them to be stigmatized by others; patients reported that others would stare, or make unkind comments or assumptions [10, 26, 28].

##### Differences Between Adult and Pediatric Patients

Figure 2 indicates the impacts reported by adult patients only, pediatric patients only, and both. Adult patients reported several physical functioning/ADL impacts that pediatric patients did not, including impacts on childcare, exercise, household chores, and shopping. Adult patients also reported feeling isolated, impact on work (reduced productivity, unable to work), and financial impacts. Adult patients also reported feeling guilt, whereas this was not observed in the pediatric studies. Pediatric patients reported specific school-related impacts, although these broadly corresponded to similar impacts for working adults, e.g., absence and unable to keep up with work.

#### Most Bothersome/Salient Aspects of FSGS

Three studies reported the most salient aspects of FSGS based on patient input [10, 11, 22]. The findings from all three studies were synthesized, although it should be noted that the three studies explored saliency differently: one asked patients to rate how bothersome they found each concept [22], another explored how long patients spent discussing each concept [10], and another analyzed which concepts were both reported frequently and had a high disturbance rating [11]. Overall, the most salient symptoms were swelling/puffiness (edema) [10, 11, 22] and fatigue [10, 11, 22]. Other salient symptoms included bloating [22], weight gain [22], sleep problems, [11, 22], dry/itchy skin [11], appetite loss [11], shortness of breath [11], and pain [10]. Impacts reported to be of greatest concern to patients included the emotional impact of the unknown and the unpredictable nature of FSGS, causing feelings of worry, anxiety, and frustration [10, 11, 22], impacts on work/school [10, 11], and impacts on social wellbeing [10, 11].

## Discussion

This targeted qualitative literature review identified a total of 13 qualitative studies and several other sources, such as patient testimonials. Data were extracted to develop a conceptual model of FSGS, including experiences of adult and pediatric patients. An existing conceptual model of FSGS was also identified in the literature and cross-checked with the findings of this literature review [11]. The conceptual model developed in this study adds to the previous understanding of patients’ experience of FSGS, and specifically includes concepts reported by pediatric patients.

The conceptual model highlights the complexity of the lived patient experience of FSGS. A conceptual model aims to provide a comprehensive depiction of patient experience; however, to inform decision-making, it is also important to understand which of these experiences are the most salient to patients. This review identified swelling (edema) and fatigue as particularly bothersome symptoms of FSGS, associated with pain, weight changes, skin problems, and a driver of stigmatization and emotional and psychological impairment. In addition, the chronic and unpredictable relapsing/remitting nature of FSGS was associated with impact on physical function and daily activities, ability to work, and feelings of anxiety, depression/sadness, and hopelessness. Notably, some of the bothersome symptoms identified in this review were also documented by the SONG-GD initiative as important patient-reported outcomes for clinical trial measurement in glomerular disease, notably fatigue, anxiety, impact on daily activities, and ability to work [13].

The primary strength of this study is that the data were obtained from multiple sources that included published literature and non-traditional sources, such as patient testimonials from patient advocacy groups such as NephCure. Inclusion of non-traditional sources provided diversity in the dataset and included the perspective of patients which may have otherwise been missed from other studies using traditional research methods. Furthermore, a clinical expert review of the conceptual model contributed to the robustness of this research.

A potential weakness of this study is that publications that included descriptions of primary NS of any cause were included because many patients with FSGS do not obtain a specific diagnosis due to the invasiveness of diagnostic kidney biopsies. However, very few concepts were identified that were reported by patients with NS alone. Additionally, the decision to include studies of patients with NS is justified by estimates that FSGS accounts for 40% of NS cases in adults [3]. Despite this, it is recognized that the specificity of the results may have been affected, and further qualitative research with patients with FSGS of all ages is required to further substantiate the signs/symptoms and impact experiences documented in the conceptual model.

Other limitations of this study are acknowledged. Beyond swelling (edema) and fatigue, which were reported in all three studies exploring saliency, there were discrepancies in the most salient/bothersome symptoms reported [10, 11, 22]. Further research is necessary to confirm the most bothersome symptoms and impacts, and thus inform assessments in future research. A further limitation was that the review eventually included only English language publications and studies conducted in North America. However, the eligibility criteria (Table 1) stipulated that any highly relevant non-English publications would be translated and extracted; in eventuality, none were identified in this review. It may therefore be concluded that research in this patient population has been primarily conducted in North America, and further research in other countries and cultures is recommended.

Further qualitative research can allow refinement and confirmation of the conceptual model. Some symptoms, such as fatigue, swelling, and pain, require further investigation, as there was variation in the literature in how these experiences were reported. For example, pain causality was associated with swelling (edema) and skin stretching but also with muscle and joint pain. The lack of understanding in experiences such as pain may limit the extent to which this conceptual model currently encompasses a specific and detailed disease experience of FSGS. Additional data may also help to delineate core disease symptoms from secondary symptoms, or treatment side effects—an attribution which is inherently difficult for patients to make—and to distinguish between proximal and distal impacts of FSGS. For example, it is expected that ability to work and study is a distal impact of FSGS, mediated by the symptom experience and impact on physical function. However, it is not clear which specific symptoms cause this impact. For patients, identifying and treating/managing these symptoms could be critical to improve their work function, and to alleviate the subsequent financial impact.

The insights gained from this study can be used to ensure that drug developers and other researchers can measure what matters to patients by informing selection and/or development of relevant COAs for inclusion in future clinical research [31]. Given the onset of symptoms in childhood, COAs that can be understood and completed by pediatric patients as well as adult patients are required to comprehensively measure the patient experience.

## Conclusion

Secondary analysis of published qualitative literature informed the development of a conceptual model describing the patient experience of FSGS. The conceptual model provides a useful tool to inform the selection and/or development of clinical outcome assessments for use in future FSGS clinical trials.

### Supplementary Information

Below is the link to the electronic supplementary material.

Supplementary file1 (PDF 193 KB)

## Data Availability

Data sharing is not applicable to this article as no datasets were generated or analyzed during the current study.
