# Supplementary material for: Development of a Conceptual Model for the Patient Experience of Focal Segmental Glomerulosclerosis (FSGS): A Qualitative Targeted Literature Review
Source: Adv Ther. 2023 Oct 11;40(12):5155–67. doi: 10.1007/s12325-023-02651-6 (PMC10611865; doi:10.1007/s12325-023-02651-6)
Supplement: Supplementary file 1 — Supplementary file1 (PDF 193 KB) [file 12325_2023_2651_MOESM1_ESM.pdf]

Supplementary Material

Development of a Conceptual Model for the Patient Experience of Focal Segmental Glomerulosclerosis (FSGS) – A Qualitative Targeted Literature Review

Authors

Natalie VJ Aldhouse MSc[1], Helen Kitchen, MSc[1], Tamara Al-Zubeidi, BSc[1], Madeleine Thursfield MA[1], Randall Winnette MSc[2], Sandi See Tai MD[3], Linda Zhu MD PhD[3], Nicolas Garnier PhD[3], Christine L Baker JD MPH MBA[2]

1 Clinical Outcomes Assessment, Clarivate, London, UK.

2 Pfizer Inc, New York, NY, USA.

3 Pfizer Inc, Collegeville, PA, USA.

Corresponding author

Natalie VJ Aldhouse; **natalie.aldhouse@clarivate.com**

Table S1. Electronic database search in Ovid MEDLINE(R) and Epub Ahead of Print, In-Process, In-Data-Review & Other Non-Indexed Citations and Daily 1946 to June 10, 2021

#	Term	Results
1	Glomerulosclerosis, Focal Segmental/ or focal segmental glomerulosclerosis.mp. or focal glomerular sclerosis.mp. or focal glomerulosclerosis.mp. or (focal adj (sclerosing or segmental or nodular) adj (glomerulosclerosis or glomerulosclerosis or glomerulonephritis or glomerulonephritides)).mp. or segmental glomerular hyalinosis.mp. or segmental hyalinosis.mp. **[FSGS terms]**	7854
2	Nephrotic Syndrome/ or nephrotic syndrome$.mp. **[Nephrotic syndrome terms]**	23507
3	exp Qualitative Research/ or qualitative.mp. or Interviews as topic/ or interview$.mp. or exp Focus Groups/ or focus group$.mp. or conceptual model$.mp. or conceptual framework$.mp. or concept mapping.mp. **[Qualitative study terms]**	629574
4	1 or 2	28749
5	4 and 3	107

Abbreviations: FSGS, focal segmental glomerulosclerosis

Table S2. Electronic database search in Ovid Embase 1980 to 2021 Week 22

#	Term	Results
1	focal glomerulosclerosis/ or focal segmental glomerulosclerosis.mp. or focal glomerular sclerosis.mp. or focal glomerulosclerosis.mp. or (focal adj (sclerosing or segmental or nodular) adj (glomerulosclerosis or glomerulosclerosis or glomerulonephritis or glomerulonephritides)).mp. or segmental glomerular hyalinosis.mp. or segmental hyalinosis.mp. **[FSGS terms]**	11508
2	exp nephrotic syndrome/ or nephrotic syndrome$.mp. **[Nephrotic syndrome terms]**	29013
3	exp qualitative research/ or qualitative.mp. or exp interview/ or interview$.mp. or focus group$.mp. or conceptual model$.mp. or conceptual framework$.mp. or concept mapping.mp. **[Qualitative study terms]**	835823
4	1 or 2	36237
5	4 and 3	209

Abbreviations: FSGS, focal segmental glomerulosclerosis

Table S3. Electronic database search in Ovid APA PsycInfo 1967 to May Week 5 2021

#	Term	Results
1	focal segmental glomerulosclerosis.mp. or focal glomerular sclerosis.mp. or focal glomerulosclerosis.mp. or (focal adj (sclerosing or segmental or nodular) adj (glomerulosclerosis or glomerulosclerosis or glomerulonephritis or glomerulonephritides)).mp. or segmental glomerular hyalinosis.mp. or segmental hyalinosis.mp. **[FSGS terms]**	16
2	nephrotic syndrome$.mp. **[Nephrotic syndrome terms]**	88
3	exp Qualitative Methods/ or qualitative.mp. or exp Interviews/ or interview$.mp. or exp Focus Group/ or focus group$.mp. or conceptual model$.mp. or conceptual framework$.mp. or concept mapping.mp. **[Qualitative study terms]**	546501
4	1 or 2	97
5	4 and 3	7

Abbreviations: FSGS, focal segmental glomerulosclerosis

Table S4. Summary of sources identified in literature review

Study name, reference	Study purpose	Study setting	Population (Condition, Age group)	Sample Size N	Ethnicity n (%)	Sex n (%)	Age (Years)
**Studies enrolling adults**							
Beanlands, 2017 (20)	To explore patient and parent perspectives on learning needs related to NS.	US & Canada	NS Adult/ Pediatric	Adults, 22<br><br>*Caregivers of children, 25*	*Adult patients:*<br><br>Caucasian, 15 (69)<br>Black, 1 (5)<br>Asian, 1 (5)<br>American Indian 1 (5)<br>Multiracial 1 (5)<br>Other 2 (9)	*Adult patients:*<br><br>Female, 11 (50)	*Adult patients:*<br><br>Median (IQR range), 52.5 (41,64)
Bressler, 2020 (21)	To share the patient voice for FSGS.	US	FSGS Adult	1	NR	Male	70
Carlozzi, 2021 (10)	To develop a patient-reported outcome assessment appropriate for use in children and adults with FSGS and MCD.	US	FSGS Adult/ Pediatric	Adults, 10<br><br>Children, 11	*Reported for the total sample only:*<br>White, 18 (85.7)<br>African American, 2 (9.5)<br>Asian, 1 (4.8)<br>Hispanic or Latino, 4 (19.0)	*Reported for the total sample only:*<br><br>Female, 11 (52.4)	NR for adult patients
English, 2019 (11)	To qualitatively examine the symptoms and impact of FSGS.	US	FSGS Adult	25	White, 15<br>Black (African American), 7<br>Asian, 2<br>Hispanic, 1	Female, 17<br>Male, 8	Range, 20–73

Study name, reference	Study purpose	Study setting	Population (Condition, Age group)	Sample Size N	Ethnicity n (%)	Sex n (%)	Age (Years)
Mathias, 2017 (22)	To develop patient-reported outcome questionnaires to measure patient experiences related to FSGS.	US	FSGS<br>Adult	30	White, 23 (77)<br>African American, 4 (13)<br>Asian, 2 (7)<br>Latino Hispanic, 1 (3)	Female, 20 (67%)	Mean (SD): 40 (11)
NephCure, 2016 (28)	To describe how NS has affected adults who live with it every day.	US	NS<br>Adult	3	NR	NR	NR
NephCure, 2021 (25)	To share the stories and experiences of adults living with FSGS.	US	FSGS<br>Adult	24	NR	NR	NR
NephCure, 2021 (24)	To share the stories and experiences of adults living with NS.	US	NS<br>Adult	5	NR	NR	NR
**Studies enrolling children, or the caregivers of children**							
Beanlands, 2017 (20)	To explore patient and parent perspectives on learning needs related to NS.	US & Canada	NS<br>Adult/<br>Pediatric	Adults, 22<br><br>Caregivers of children, 25	*NR for the child patients or for their caregivers*	*NR for the child patients or for their caregivers*	*NR for the child patients or for their caregivers*

Study name, reference	Study purpose	Study setting	Population (Condition, Age group)	Sample Size N	Ethnicity n (%)	Sex n (%)	Age (Years)
Carlozzi, 2021 (10)	To develop a patient-reported outcome assessment appropriate for use in children and adults with FSGS and MCD.	US	FSGS Adult/ Pediatric	Adults, 10<br><br>Children, 11	*Reported for the total sample only:*<br>White, 18 (85.7)<br>African American, 2 (9.5)<br>Asian, 1 (4.8)<br>Hispanic or Latino, 4 (19.0)	*Reported for the total sample only:*<br><br>Female, 11 (52.4)	n (% of total sample)<br><br>8-13, 5 (23.8)<br>14-17, 6 (28.6)
NephCure, 2016 (29)	To describe how NS and the myriad treatments can greatly impact the day to day life, self image, and emotional well-being of three children who live with pediatric NS.	US	NS Pediatric	3	NR	NR	NR
NephCure, 2021 (27)	To share the stories and experiences of children living with FSGS.	US	FSGS Pediatric	18	NR	NR	NR
NephCure, 2021 (26)	To share the stories and experiences of children living with NS.	US	NS Pediatric	16	NR	NR	NR
Vance, 1983 (23)	To examine the effect of nephrotic syndrome on various aspects of behavior and development in a group of pediatric patients.	US	NS Pediatric	43	White, (79.1)	Male, (54.5)	Mean (SD): 11.6 (4.4)

Abbreviations: FSGS, focal segmental glomerulosclerosis; IQR, inter-quartile range; NR, not reported; NS, nephrotic syndrome; SD, standard deviation; US, United States

Data are reported as presented in the publications; if percentages were not included, they have not been calculated here.
